# Expression of CCL2 and CCR2 in the hippocampus and the interventional roles of propofol in rat cerebral ischemia/reperfusion

**DOI:** 10.3892/etm.2014.1757

**Published:** 2014-06-04

**Authors:** YONG-QING GUO, LI-NA ZHENG, JIAN-FENG WEI, XIAO-LAI HOU, SHU-ZHEN YU, WEI-WEI ZHANG, JIAN-MIN JING

**Affiliations:** Department of Anesthesiology, Shanxi Provincial People’s Hospital, Taiyuan, Shanxi 030012, P.R. China

**Keywords:** chemokine ligand 2, chemokine receptor type 2, cerebral ischemia/reperfusion, hippocampus

## Abstract

The aim of the present study was to determine the roles of the chemotactic factor, chemokine ligand 2 (CCL2), and its receptor, chemokine receptor type 2 (CCR2), in the hippocampus of rats with cerebral ischemia/reperfusion injury. In total, 24 Sprague-Dawley rats, weighting 250–300 g, were randomly divided into three groups (n=8): Sham-operated (C group), cerebral ischemia/reperfusion injury (I/R group) and propofol-intervention (P group) groups. The rats were sacrificed at 6 h after the ischemia/reperfusion surgery, and the brains were obtained to isolate the hippocampus. The mRNA expression levels of CCL2 and CCR2 in the hippocampus were analyzed by quantitative polymerase chain reaction, while the protein expression levels of CCL2 and CCR2 were determined by western blot analysis. The expression levels of CCL2 and CCR2 in the procerebrum were markedly elevated in the I/R and P groups at 6 h after the ischemia/reperfusion surgery when compared with the C group (P<0.05). In addition, the mRNA expression levels of CCL2 and CCR2 decreased significantly in the P group as compared with that in the I/R group (P<0.05). Therefore, CCL2 and CCR2 may be involved in the mechanisms underlying cerebral ischemia/reperfusion injury, and propofol may protect the brain through regulating the expression of CCL2 and CCR2.

## Introduction

Chemotactic factors are a class of cytokines that cause the migration of special receptor-expressing cells, playing an important role in the inflammatory reaction. Chemokine ligand 2 (CCL2), which was previously known as monocyte chemoattractant protein-1, is a monocyte chemotactic factor. CCL2 can specifically bind to its receptor, chemokine receptor type 2 (CCR2), to regulate the migration and infiltration of monocytes, T cells and natural killer cells in the inflammatory area (1–3). CCR2, a G protein-coupled receptor, is widely expressed on endothelial cells, horizontal cells, gitter cells and neurons in the central nervous systems of a number of species in addition to humans (4–7). Furthermore, CCL2 and CCR2 have been demonstrated to be expressed in numerous encephalic regions in addition to the hippocampus (6–8). The expression pattern of CCR2 can be altered in a neuropathological state.

Cerebral ischemia is often accompanied with reperfusion injury, thus, therapy targeted to reduce the damage is urgently required in clinical practice. Since there is no clinically effective method to treat cerebral ischemia/reperfusion injury to date, it is necessary to further study this disease. The selection of anesthetic drugs for patients with cerebral ischemia in the perioperative period requires careful consideration by anesthesiologists. Propofol is a common anesthetic drug used widely as a clinical anesthesia due to the numerous advantages, including rapid action, quick clearance and fewer adverse side effects.

A number of studies have demonstrated that propofol exhibits protective roles in the brain, however, the majority of studies have been conducted with ischemia models (9,10). Propofol has also been reported to exhibit a potential protective role in procerebral ischemia/reperfusion injury (11), however, the exact mechanism remains unclear. In the present study, the effects of preadministration of propofol on the expression levels of CCL2 and CCR2 in the hippocampus were investigated in rats with procerebral ischemia/reperfusion injury. The aim of the study was to provide more useful evidence for the further study of cerebral ischemia/reperfusion injury.

## Materials and methods

### Animals and grouping

In total, 24 healthy adult male Sprague-Dawley rats, weighing 250–300 g, were obtained from the Physiological Laboratory Animal Center at Shanxi Medical University (Taiyuan, China). The study was conducted in strict accordance with the recommendations in the Guide for the Care and Use of Laboratory Animals of the National Institutes of Health, and the animal use protocol was reviewed and approved by the Institutional Animal Care and Use Committee of Shanxi Provincial People’s Hospital (Taiyuan, China). Animals were housed in a specific pathogen-free room at a constant temperature of 25°C and a relative humidity of 45%. All the animals received humane care in compliance with the strict guiding principles of the National Institution of Health for Experimental Care and Use of Animals. The experimental design and procedures were approved by the Institutional Ethical Committee for Animal Care and Use of Shanxi Provincial People’s Hospital.

Rats were randomly divided into three groups (n=8): Sham-operated (C group), cerebral ischemia/reperfusion injury (I/R group) and propofol-intervention groups (P group). Rats in the sham-operated group received all the surgical procedures, but without the bloodletting and reinfusing process. In the P group, a dosage of 50 mg/kg propofol (FJ261, AstraZeneca, Milan, Italy) was injected into the vena femoralis of the rats prior to the cerebral ischemia surgery. Rats in the I/R group were administered 1 ml physiological saline instead of propofol by vena femoralis injection prior to the cerebral ischemia surgery.

### I/R protocol

Rats were anesthetized with 10% chloral hydrate (0.3 ml/kg, i.p.) and fixed in a supine position. Following a median incision of the neck skin, the bilateral common carotid artery was carefully dissected and exposed. The left femoral artery and vein were cannulated with NO.24 trochar. The arterial blood pressure was monitored continuously using a BL-420F Bio-function Experimental System (Thai Meng Technology Co., Ltd., Chengdu, China). At 15 min prior to bloodletting, the venous blood gas was measured periodically (AVL-939 mini blood gas pH analyzer; Switzerland) to maintain the pH, arterial carbon dioxide tension (PaCO_2_) and arterial oxygen tension (PaO_2_) at normal ranges (pH, 7.35–7.45; PaCO_2_, 35–45 mmHg; PaO_2_, 290–140 mmHg), where 1 kPa is equal to 7.5 mmHg. Next, the rats were bled slowly over 5 min from the left femoral vein by withdrawing or infusing blood into a 10 ml heparinized syringe to maintain the mean arterial pressure between 35 and 45 mmHg. Immediately after reaching 35 mmHg, the common carotid arteries were occluded using atraumatic aneurysm clamps for 10 min to induce procerebral ischemia. The brain circulation was restored by unclamping the common carotid arteries and reinfusing the blood into the rat for 5 min. The trochars were removed from the femoral artery and vein to terminate the reperfusion.

### Sampling

Rats were sacrificed by decapitation at 6 h after reperfusion and anesthesia. The brain was removed and the bilateral hippocampus was separated immediately on plates at −20°C. The hippocampi collected were frozen quickly in liquid nitrogen and stored at −80°C.

### Quantitative polymerase chain reaction (qPCR)

For qPCR, 50-mg samples of hippocampal tissue were disrupted in 1 ml TRIzol reagent (Invitrogen Life Technologies, Carlsbad, CA, USA) to form a homogenate. Next, the homogenates were treated with chloroform, isopropyl alcohol and ethanol to extract the total RNA. cDNA synthesis was conducted on the RNA product using a PrimeScript RT reagents kit (Takara Bio, Inc., Shiga, Japan), according to the manufacturer’s instructions. PCR was performed in a total volume of 50 μl (containing 3 μl cDNA, 50 pmol forward and reverse primers, 0.4 μmol/l dNTP, 5 μl 10X buffer and 2.5 IU *Taq* polymerase) using a Gradient PCR machine (Biometra GmbH, Göettingen, Germany). The following gene-specific primer pairs were used: CCL2, forward, 5′-CTG TCT CAG CCA GAT GCA GTT-3′ and reverse, 5′-GAG CTT GGT GAC AAA TAC TAC A-3′; CCR2 forward, 5′-GTT CTC TTC CTG ACC ACC TTC-3′ and reverse, 5′-CTT CGG AAC TTC TCA CCA ACA-3′; β-actin forward, 5′-TCC CTC AAG ATT GTC AGC AA-3′ and reverse, 5′-AGA TCC ACA ACG GAT ACA TT-3′. The product sizes were 147, 157 and 308 bp, respectively. All the samples were run in cycles as follows: One cycle of 94°C for 3 min; 35 cycles of 94°C for 30 sec, 50°C for 30 sec and 72°C for 1 min; one cycle of 72°C for 5 min. The samples were then cooled to 4°C.

The final PCR products were run on 2% agarose gel, stained with ethidium bromide and observed using a viltalight lamp. A DNA marker with an 100-bp ladder was used to identify the product sizes. The stained DNA bands were then analyzed with a computer gel image analysis system (Kodak, Rochester, NY, USA). The density of the PCR products was calculated using the area under the curves, while the relative mRNA expression levels of CCL2 and CCR2 were determined using the ratio of the density of CCL2 or CCR2 against that of β-actin.

### Western blot analysis

Total protein of the hippocampal tissue was extracted using a protein extraction kit (Apply Gen Technologies, Inc., Beijing, China). The proteins were separated with SDS-PAGE and transferred onto polyvinylidene fluoride membranes for immunoblotting. Next, 10% defatted milk powder dissolved in Tris-buffered saline Tween-20 (TBST) was added to the membranes to block the endogenous horseradish peroxidase for 1 h in room temperature. The membranes were then incubated with mouse anti-rat CCL2, CCR2 and β-actin antibodies (1:1,000; Santa Cruz Biotechnology, Inc., Santa Cruz, CA, USA) overnight at 4°C. Following washing with TBST three times, each time for 10 min, the membranes were incubated with horseradish peroxidase-conjugated anti-mouse IgG (1:10,000; Beijing Zhongshan Golden Bridge Biotechnology Co., Ltd., Beijing, China) for 2 h at room temperature. The specific protein bands on the membranes were visualized using an enhanced chemiluminescence kit, according to the manufacturer’s instructions. The bands were scanned using a Gel-imaging system (Bio-Rad 2000; Bio-Rad, Hercules, CA, USA) and analyzed using Quantity One software. The optical density values of the two targeted proteins were calibrated against that of β-actin, from which the protein expression levels of CCL2 and CCR2 were calculated.

### Statistical analysis

Statistical analysis was performed with the SPSS software program v 11.0 (SPSS, Inc., Chicago, IL, USA). Data are expressed as the mean ± standard deviation. Differences among the groups were compared using one-factor analysis of variance, where P<0.05 was considered to indicate a statistically significant difference.

## Results

### Relative mRNA expression levels of CCL2 and CCR2 in rats

**Figure 1:**
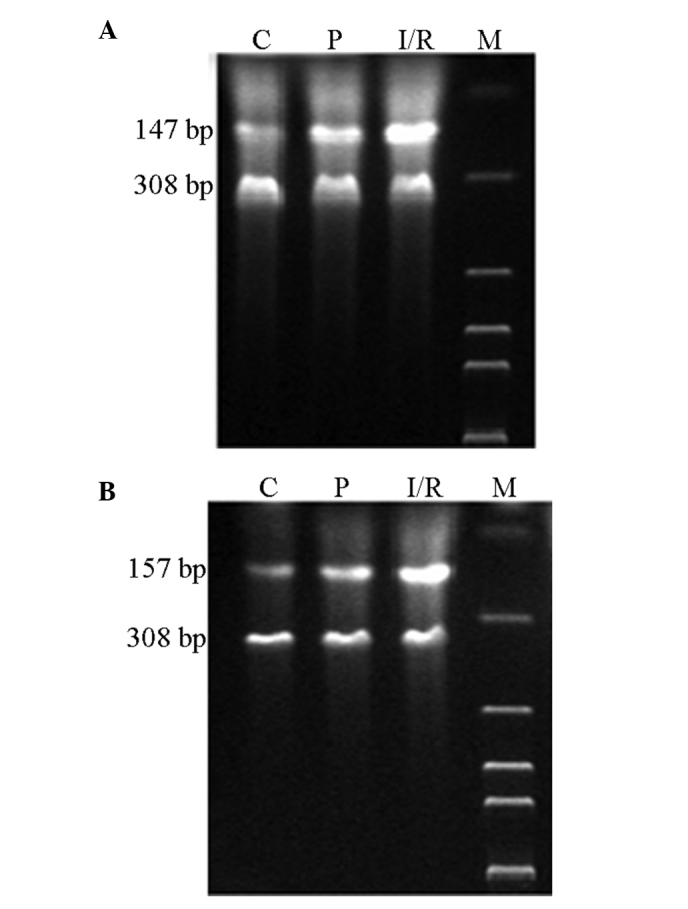
mRNA expression levels of (A) CCL2 and (B) CCR2 were detected by qPCR. CCL2, chemokine ligand 2; CCR2, chemokine receptor type 2; qPCR, quantitative polymerase chain reaction; C, sham-operated group; P, propofol-intervention group; I/R, cerebral ischemia reperfusion injury group; M, DNA marker.

Expression levels of CCL2 and CCR2 mRNA in the hippocampus were analyzed by qPCR following cerebral ischemia/reperfusion. The mRNA expression levels of CCL2 and CCR2 in the hippocampus neurons of the I/R and P groups increased significantly (P<0.05) when compared with the expression levels in the C group. However, the expression levels of CCL2 and CCR2 in the hippocampus neurons of the P group decreased markedly (P<0.05) when compared with the I/R group (Table I; Fig. 1).

### Protein expression levels of CCL2 and CCR2 in rats

**Figure 2:**
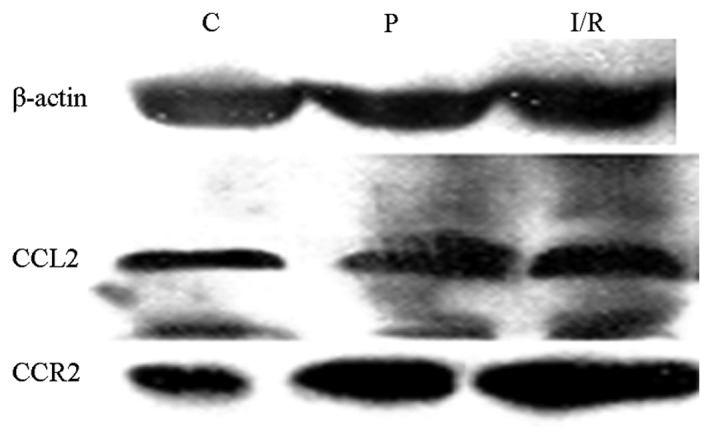
Protein expression levels of CCL2 and CCR2 were determined by western blot analysis. CCL2, chemokine ligand 2; CCR2, chemokine receptor type 2; C, sham-operated group; P, propofol-intervention group; I/R, cerebral ischemia reperfusion injury group.

Protein expression levels of CCL2 and CCR2 in the hippocampus were analyzed by western blot analysis following cerebral ischemia/reperfusion. The protein expression levels of CCL2 and CCR2, as detected by western blot analysis, are shown in Fig. 2. Expression levels of CCL2 and CCR2 in the I/R and P groups were significantly higher compared with those in the C group. In addition, CCL2 and CCR2 expression levels in the P group decreased markedly when compared with those in the I/R group (Table II).

## Discussion

Preliminary investigations demonstrated that the mRNA expression levels of CCL2 in the brain cortex increased significantly at 2 h after ischemia in an ischemia/reperfusion model. Expression levels peaked at 16 h after reperfusion and were maintained at a high level for 48 h following reperfusion. Combining the results from the preliminary experiments, in the present study, the expression levels of CCL2 and CCR2 were investigated in rat hippocampal neurons at 6 h after reperfusion. In addition, the present study investigated the protective roles of propofol on the brain by injecting propofol into the vena femoralis prior to ischemia surgery.

The neuroprotective effect of propofol is the result of a number of mechanisms, including reducing the brain oxygen metabolism rate, removing the oxygen free radicals (12,13), activating the c-aminobutyric acid type A receptor (14), inhibiting the glutamate receptors (15), reducing the extracellular glutamate concentration (16) and increasing the glutamic acid salt intake (17) via inhibiting the release of glutamate-dependent Na^+^ channels. In the present study, propofol was shown to significantly downregulate the expression levels of CCL2 and CCR2 in the hippocampal tissues, indicating that propofol may decrease the inflammatory reactions caused by ischemia during reperfusion. Propofol may exhibit this neuroprotective effect in cerebral ischemia/reperfusion injury by regulating the expression levels of CCL2 and CCR2.

Clinically, anesthetic drugs are often administered prior to the occurrence of brain ischemia. Models can imitate cerebral ischemia caused by acute hemorrhage, cardiac arrest and certain shock (18). Therefore, future research on the mechanisms underlying hypoxic-ischemic brain damage should investigate the effects of CCL2 and CCR2 using rats with cerebral ischemia/reperfusion.

Previous research with regard to stroke has also demonstrated that overexpression of CCL2 can aggravate ischemic brain injury, and inhibiting the expression of CCL2 can reduce injury. Inflammatory reactions are known to play important roles in brain injury (19). In axon injury, the CCL2 that is produced by early glial cells causes an intrinsic reaction, inducing leukocyte infiltration. In addition, CCL2 can supplement T-cell activation-3/CCL1, the ligand of the CCR8 receptor, to guide the infiltration of T cells in encephalomyelitis (20). Thus, chemotactic factors derived from glial cells can be used as key regulators to accommodate the central immune system (21). However, CCR2 and CCL2 are critical for the attraction of leukocyte infiltration (22), and the interaction between them also influences the processing and clearance of cerebral hemorrhage (23–24).

CCR2 is widely expressed on brain neurons, astrocytes and gitter cells, and is a high-affinity receptor for CCL2. When CCL2 binds to its receptor, monocytes are caused to migrate to the inflammatory sites and participate in the inflammatory reactions mediated by monocytes. The results of the present study indicated that there was a synchronous increase in the expression levels of CCL2 and CCR2 in cerebral ischemia/reperfusion injury and a synchronous decrease when propofol was injected into the rats prior to surgery. These observations indicate that preadministration of propofol may suppress the inflammatory reaction by regulating the expression levels of CCL2 and CCR2, which then consequently reduces the danger of procerebral ischemia/reperfusion injury in rats.

However, only one time point and single dosage were analyzed in the present study. Data with more time points and multiple administration concentrations, as well as morphological evidence, may supplement these results in the future.

In conclusion, CCL2 and CCR2 are involved in the pathogenic mechanisms underlying cerebral ischemia/reperfusion injury in rats, and preadministration of propofol can suppress the expression of CCL2 and CCR2. In-depth study to investigate the exact roles of chemotactic factors in immunological injury of the central nervous system may be of great importance to improve the prognosis of cerebral ischemia/reperfusion injury and identify specific novel therapeutic targets.

## Figures and Tables

**Table I:** Relative mRNA expression levels of CCL2 and CCR2.

Group	CCL2	CCR2
C	0.49±0.27	0.29±0.13
I/R	1.58±0.42^a^	0.56±0.21^a^
P	0.76±0.29^b^	0.47±0.22^b^

a P<0.01, vs. C group;

b P<0.05, vs. I/R group. Results are expressed as the mean ± standard deviation (n=8).

CCL2, chemokine ligand 2; CCR2, chemokine receptor type 2; C, sham-operated; P, propofol-intervention; I/R, cerebral ischemia reperfusion injury.

**Table II:** Protein expression levels of CCL2 and CCR2.

Group	CCL2	CCR2
C	0.21±0.016	0.23±0.023
I/R	0.79±0.072^a^	0.47±0.085^a^
P	0.56±0.029^b^	0.33±0.069^b^

a P<0.01, vs. C group;

b P<0.05, vs. I/R group. Results are expressed as the mean ± standard deviation (n=8).

CCL2, chemokine ligand 2; CCR2, chemokine receptor type 2; C, sham-operated; P, propofol-intervention; I/R, cerebral ischemia reperfusion injury.
